# FLP-4 neuropeptide and its receptor in a neuronal circuit regulate preference choice through functions of ASH-2 trithorax complex in *Caenorhabditis elegans*

**DOI:** 10.1038/srep21485

**Published:** 2016-02-18

**Authors:** Yonglin Yu, Lingtong Zhi, Xiangmin Guan, Daoyong Wang, Dayong Wang

**Affiliations:** 1 Key Laboratory of Developmental Genes and Human Disease in Ministry of Education, Medical School, Southeast University, Nanjing 210009, China

## Abstract

Preference choice on food is an important response strategy for animals living in the environment. Using assay system of preference choice on bacterial foods, OP50 and PA14, we identified the involvement of ADL sensory neurons in the control of preference choice in *Caenorhabditis elegans*. Both genetically silencing and *ChR2*-mediated activation of ADL sensory neurons significantly affected preference choice. ADL regulated preference choice by inhibiting function of G protein-coupled receptor (GPCR)/SRH-220. ADL sensory neurons might regulate preference choice through peptidergic signals of FLP-4 and NLP-10, and function of FLP-4 or NLP-10 in regulating preference choice was regulated by SRH-220. FLP-4 released from ADL sensory neurons further regulated preference choice through its receptor of NPR-4 in AIB interneurons. In AIB interneurons, NPR-4 was involved in the control of preference choice by activating the functions of ASH-2 trithorax complex consisting of SET-2, ASH-2, and WDR-5, implying the crucial role of molecular machinery of trimethylation of histone H3K4 in the preference choice control. The identified novel neuronal circuit and the underlying molecular mechanisms will strengthen our understanding neuronal basis of preference choice in animals.

Animals’ decision making includes two main levels of complexity, behavioral choice and value-based decision making^1^,^2^. Decision making may be helpful for maximizing the evolutionary fitness in an unpredictable world. Behavioral choice concerns proximal causes for the selection between alternative sensory cues^2^. Nematode *Caenorhabditis elegans* is an attractive model animal for behavioral study due to its simple nervous system and ability to sense diverse environmental stimuli, such as touch, smell, taste and temperature^3^,^4^. Simplicity and genetic tractability of *C. elegans* nervous system makes it a useful assay system for seeking the biological mechanisms of behavioral choice^5^,^6^. *C. elegans* can exhibit several types of behavioral choice based on its response to paired or multiple stimuli, such as attractants *versus* aversive stimuli or different attractants^7^,^8^,^9^,^10^,^11^,^12^.

In *C. elegans*, olfactory chemotaxis towards food-associated odors is an innate behavior, and highly reproducible among animals^13^. Preference choice on bacterial food is one of the assayed behavioral choices in *C. elegans*^12^,^14^. In this behavioral choice assay system, nematodes will migrate towards one of the two examined bacterial lawns on opposite sides in a plate (Fig. 1a)^12^,^14^. Usually a harmless bacterium such as OP50 and a pathogenic bacterium such as PA14 are used in the preference choice assay system^12^,^15^. Previous study has implied that the deficits in differentiation of AWB or AWC sensory neurons, required for the olfactory perception, affect preference choice in nematodes^14^. In *C. elegans*, AWB and AWC sensory neurons are necessary for innate preference for *Pseudomonas aeruginosa*^16^, and neurotransmitters and neuropeptides used by AWB or AWC sensory neurons may control this innate odor preference^17^. Nevertheless, neuronal circuit involved in the control of preference choice on bacterial food is still largely unclear. In addition, only limited information is available for the molecular basis of preference choice on bacterial food in *C. elegans*.

Among the well-known 12 kinds of amphid neurons (AWA, AWB, AWC, AFD, ASE, ADF, ASG, ASH, ADL, ASI, ASJ, and ASK) in *C. elegans*, ASH and ADL are important sensory neurons functioning to sense and mediate avoidance of noxious environmental stimuli, such as Cu^2+^ ion and garlic^18^,^19^,^20^. In this study, using quantitative behavior assays, and genetic and neuronal manipulations, we determined the possible roles of these sensory neurons in regulating preference choice on bacterial food and the underlying neuronal and molecular mechanisms in *C. elegans*. The new identified neuronal circuit and the related underlying mechanism for preference choice on bacterial food will strengthen our understanding neuronal basis of behavioral choice in animals.

## Results

### Genetically silencing ADL sensory neurons altered preference choice of nematodes on bacterial food

**Figure 1:**
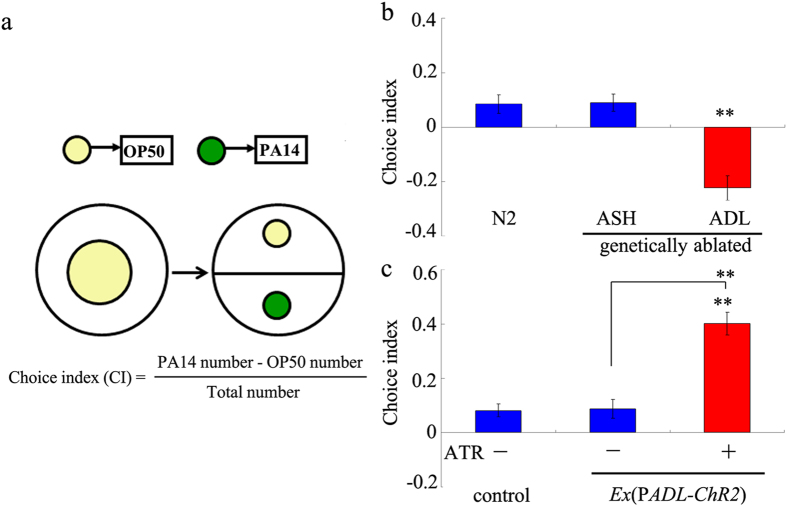
Preference choice on bacterial food in nematodes with genetically ablated or optogenetically activating ADL sensory neurons. (**a**) Schematic representation of assay system for preference choice on bacterial food. (**b**) Preference choice on bacterial food in nematodes with genetically ablated ADL or ASH sensory neurons. (**c**) Preference choice on bacterial food in nematodes with optogenetical activation of ADL sensory neurons. Optogenetical activation was performed under the *lite-1* mutation background. Control, *lite-1(ce314)*. Bars represent means ± S.E.M. ^**^P < 0.01 *vs* N2 (if not specifically indicated).

To identify candidate sensory neurons involved in the control of preference choice on bacterial food, we employed the strain with genetically silencing ASH or ADL sensory neurons. In the strain of ASH::TeTx, TeTx was specifically expressed in ASH sensory neurons using a FLP–FRT site-specific recombination system to permanently block the ASH neurotransmission^20^. *egl-1* gene encodes a cell-death activator , and can be used to genetically ablating specific neurons^21^. We obtained a strain in which ADL sensory neurons were genetically ablated by expressing *egl-1* under the control of *srh-220* promoter. Interestingly, genetically ablating ASH sensory neurons did not significantly affect preference choice compared with wild-type N2 (Fig. 1b). In contrast, we found that genetically ablating ADL sensory neurons significantly increased choice index in the preference choice assay system (Fig. 1b). Meanwhile, nematodes with genetically ablated ASH or ADL sensory neurons had similar leaving behavior from OP50 or PA14 lawns and chemotaxis to OP50 compared with wild-type N2 (Fig. S1).

### Optogenetically activating ADL sensory neurons affected preference choice of nematodes on bacterial food

To further confirm the role of ADL sensory neurons in regulating preference choice, we performed optogenetic manipulation of ADL sensory neurons. All the optogenetic manipulations of ADL sensory neurons were performed under the *lite-1* mutation background in order to eliminate the intrinsic photophobic response of *C. elegans*^22^. The *lite-1(ce314)* mutant had the similar preference choice to wild-type (data not shown). We observed that nematodes with channelrhodopsin-2 (*ChR2*)-mediated activation of ADL sensory neurons on assay plates with all-*trans* retinal (ATR) exhibited significantly enhanced choice index compared with wild-type N2 (Fig. 1c). Preference choice phenotype in nematodes with *ChR2*-mediated activation of ADL sensory neurons was opposite to that in nematodes with genetically ablated ADL sensory neurons (Fig. 1b,c). The *lite-1(ce314)* mutant had the similar leaving behavior from OP50 or PA14 lawns and chemotaxis to OP50 to wild-type (data not shown). Meanwhile, nematodes with optogenetically activating ADL sensory neurons showed the similar leaving behavior from OP50 or PA14 lawns and chemotaxis to OP50 compared with wild-type N2 (Fig. S1). These results suggest the crucial role of ADL sensory neurons in the control of preference choice of nematodes on bacterial food.

### Expression of ARR-1 in ADL sensory neurons was required for preference choice control

In *C. elegans, arr-1* gene encodes the only G protein-coupled receptor (GPCR) adaptor protein, and plays important roles in both longevity and immunity^23^. We further observed that loss-of-function mutation of *arr-1* gene caused the deficit in preference choice behavior compared with wild-type N2 (Fig. S2a). *arr-1(ok401)* mutant nematodes had similar leaving behavior from OP50 or PA14 lawns and chemotaxis to OP50 to those in wild-type N2 (Fig. S2b–S2d). Moreover, we found that expression of *arr-1* gene under the control of *srh-220* promoter rescued the deficit in preference choice in *arr-1(ok401)* mutant (Fig. S2a), suggesting that ARR-1 in ADL sensory neurons may be involved in the control of preference choice.

### GPCRs in ADL sensory neurons regulated the preference choice behavior

**Figure 2:**
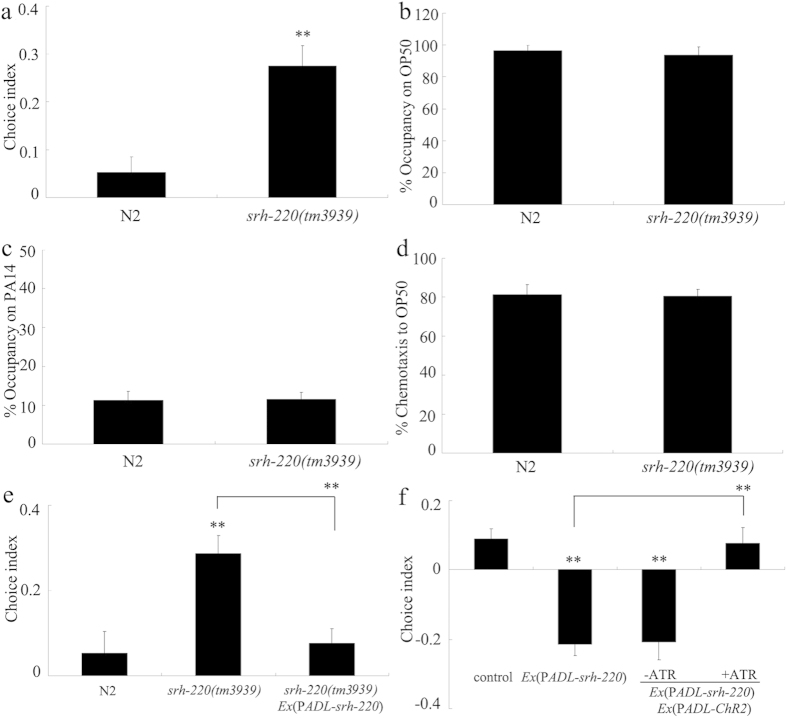
Roles of SRH-220/GPCR in ADL sensory neurons in the control of preference choice in nematodes. (**a**) Effects of *srh-220* mutation on preference choice. (**b**–**d**) Leaving behavior from bacterial lawns and chemotaxis to OP50 in *srh-220* mutants. (**e**) Rescue assay of preference choice phenotype in *srh-220* mutants. (**f**) Optogenetically activating ADL sensory neurons suppressed the function of SRH-220 in regulating preference choice. Optogenetical activation was performed under the *lite-1* mutation background. Control, *lite-1(ce314)*. Bars represent means ± S.E.M. ^**^P < 0.01 *vs* N2 (if not specifically indicated).

In *C. elegans*, some genes encoding GPCRs such as SRE-1, SRI-51, SRH-132, and SRH-220 are expressed in ADL sensory neurons^24^,^25^,^26^. Among these genes, we found that loss-of-function mutation of *srh-220* gene resulted in enhanced choice index compared with wild-type N2 (Fig. 2a). *srh-220(tm3939)* mutants had normal leaving behavior from OP50 or PA14 lawns and chemotaxis to OP50 (Fig. 2b–d). The deficit in preference choice in *srh-220(tm3939)* could be rescued by the expression of *srh-220* under the control of its own promoter (Fig. 2e).

In *C. elegans*, we further observed that overexpression of *srh-220* gene led to the significantly decreased choice index compared with wild-type N2 (Fig. 2f). Moreover, *ChR2*-mediated activation of ADL sensory neurons inhibited the preference choice phenotype in nematodes overexpressing *srh-220* gene (Fig. 2f), implying that activation of ADL may antagonize the function of GPCR/SRH-220 in regulating preference choice in nematodes.

### Signaling from ADL sensory neurons in the modulation of preference choice was primarily peptidergic

**Figure 3:**
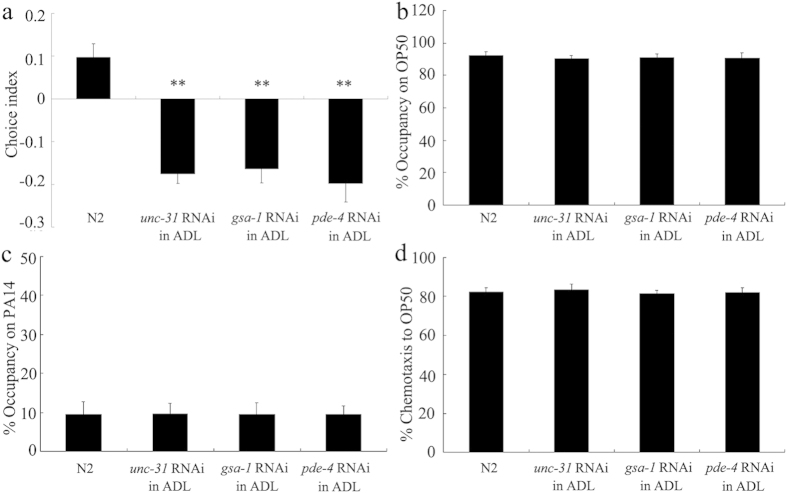
Effects of ADL-specific RNAi of *unc-31, gsa-1*, or *pde-4* gene on preference choice. (**a**) Preference choice of nematodes with ADL-specific RNAi of *unc-31, gsa-1*, or *pde-4* gene. (**b**–**d**) Leaving behavior from bacterial lawns and chemotaxis to OP50 of nematodes with ADL-specific RNAi of *unc-31, gsa-1*, or *pde-4* gene. Bars represent means ± S.E.M. ^**^P < 0.01 *vs* N2.

Previous studies have suggested that signaling from ADL sensory neurons in the modulation of ASH-mediated aversive response is primarily peptidergic^27^,^28^. We next examined the role of pathways modulating peptide release from ADL sensory neurons by neuron-specific RNA interference (RNAi). In *C. elegans, unc-31* gene encodes a DAG binding protein that plays a key role in dense core vesicle (DCV) release^29^. ADL RNAi knockdown of *unc-31* gene significantly decreased choice index compared with wild-type N2 (Fig. 3a). *gsa-1* gene encoded Ga_s_ enhances exocytosis from DCVs, and *pde-4* gene encodes a phosphodiesterase, that is predicted to alter cAMP levels^28^,^29^. Similarly, ADL RNAi knockdown of *gsa-1* or *pde-4* gene significantly decreased choice index compared with wild-type N2 (Fig. 3a). Nematodes with ADL RNAi knockdown of *unc-31, gsa-1* or *pde-4* gene showed similar leaving behavior from OP50 or PA14 lawns and chemotaxis to OP50 to those in wild-type N2 (Fig. 3b–d). These results imply that the signaling from ADL sensory neurons may be primarily peptidergic with respect to the control of preference choice in nematodes. *gsa-1(ce94)* is a gain-of-function mutant, and *pde-4(ok1290)* is a loss-of-function mutant. We further found that the *gsa-1(ce94)* mutant had the increased choice index, whereas the *pde-4(ok1290)* mutant has the decreased choice index compared with wild-type N2 (Fig. S3).

### Neuropeptides in ADL sensory neurons were involved in the control of preference choice

**Figure 4:**
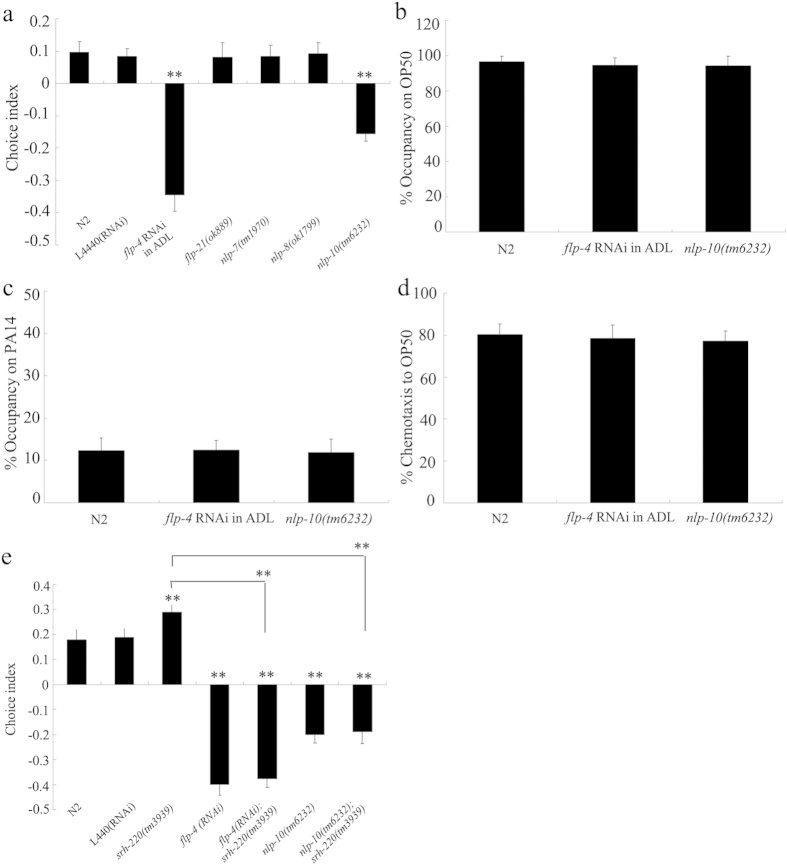
Identification of neuropeptides involved in the control of preference choice in nematodes. (**a**) Effects of genes encoding neuropeptides expressed in ADL sensory neurons on preference choice. (**b**–**d**) Effects of *flp-4* or *nlp-10* gene on leaving behavior from bacterial lawns and chemotaxis to OP50. (**e**) Genetic interaction between *srh-220* and *flp-4* or *nlp-10* in regulating preference choice. Bars represent means ± S.E.M. ^**^P < 0.01 *vs* N2 (if not specifically indicated).

In *C. elegans*, some neuropeptides were expressed in ADL sensory neurons, and these neuropeptides are encoded by *flp-4, flp-21, nlp-7, nlp-8*, and *nlp-10* genes^30^. Among these genes, we found that ADL-specific RNAi knockdown of *flp-4* or mutation of *nlp-10* significantly decreased choice index compared with wild-type N2 (Fig. 4a). Nematodes with ADL-specific RNAi knockdown of *flp-4* and *nlp-10(tm6232)* mutants had normal leaving behavior from OP50 or PA14 lawns and chemotaxis to OP50 (Fig. 4b–d). Therefore, some neuropeptides in ADL sensory neurons may be required for the control of preference choice in nematodes.

### Genetic interaction of SRH-220 with FLP-4 or NLP-10 in regulating preference choice

Moreover, we investigated the genetic interaction between *srh-220* and *flp-4* or *nlp-10* in regulating preference choice in nematodes. We found that the preference choice phenotype in double mutant of *flp-4(RNAi);srh-220(tm3783)* was similar to that in *flp-4(RNAi)* nematodes (Fig. 3e). Similarly, the preference choice phenotype in double mutant of *nlp-10(tm6232);srh-220(tm3783)* was similar to that in *nlp-10(tm6232)* mutant nematodes (Fig. 3e). Taken together, these results suggest that *flp-4* or *nlp-10* mutation may suppress the preference choice phenotype in *srh-220* mutants in nematodes.

### Functional analysis of NPR-4, receptor of FLP-4, in regulating preference choice

**Figure 5:**
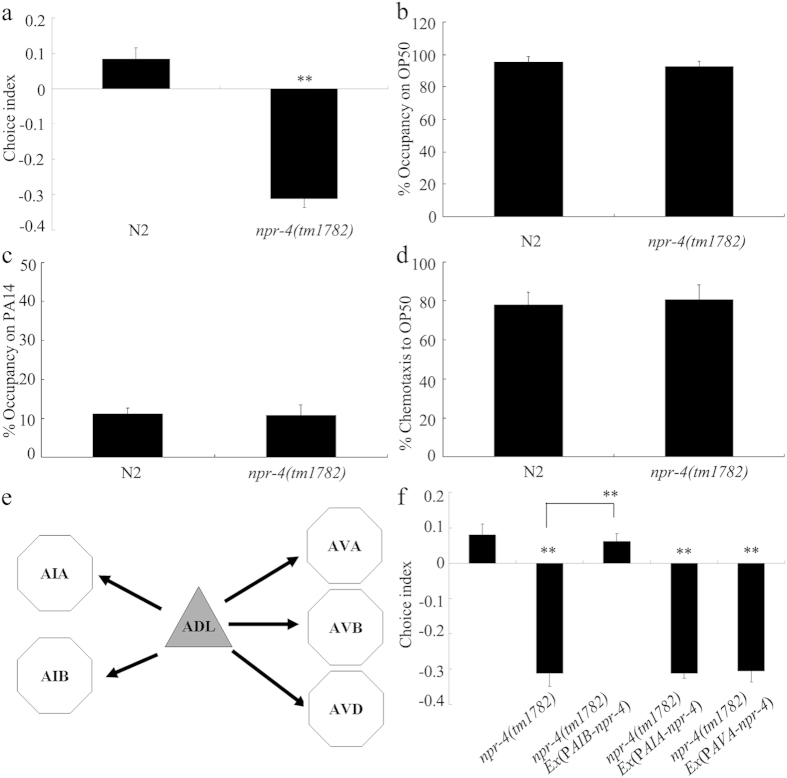
NPR-4 regulated the preference choice in nematodes. (**a**) Mutation of *npr-4* gene altered preference choice. (**b**–**d**) Mutation of *npr-4* gene did not influence leaving behavior from bacterial lawns and chemotaxis to OP50. (**e**) Schematic diagram for putative neural networks associated with ADL sensory neurons, extracted from the complete circuit^3^. Sensory neurons are shown as triangles and interneurons as hexagons. Arrows indicate chemical synapses. (**f**) Expression of NPR-4 in AIB interneurons rescued the deficit in preference choice in *npr-4* mutants. Bars represent means ± S.E.M. ^**^P < 0.01 *vs* N2 (if not specifically indicated).

In *C. elegans*, the receptor for the neuropeptide of FLP-4 is NPR-4, and the corresponding receptor for the neuropeptide of NLP-10 is unknown^30^. Loss-of function mutation of *npr-4* gene significantly decreased choice index compared with wild-type N2 (Fig. 5a). Meanwhile, *npr-4(tm1782)* mutants had normal leaving behavior from OP50 or PA14 lawns and chemotaxis to OP50 (Fig. 5b–d). These results suggest that FLP-4, together with its receptor of NPR-4, may be involved in the control of preference choice in nematodes.

In *C. elegans*, sensory inputs can be released from ADL sensory neurons to AIA, AIB, AVA, AVB, or AVD interneurons^3^ (Fig. 5e). Among AVA, AVB, and AVD interneurons involved in the control of locomotion behavior^10^, NPR-4 was reported to be able to be expressed in AVA interneurons^31^. However, expression of NPR-4 in AVA interneurons did not recover deficit in preference choice in *npr-4(tm1782)* mutants (Fig. 5f). Considering the crucial role of sensory interneurons in the control of behavioral choice^6^,^9^, we also examined the function of NPR-4 in AIA or AIB interneurons in regulating preference choice. Interestingly, we found that expression of NPR-4 in AIB interneurons could obviously rescue the deficit in preference choice in *npr-4(tm1782)* mutants (Fig. 5f). In *C. elegans*, NPR-4 could be expressed in AIB interneurons (Fig. S4).

### Mutation of *npr-4* gene altered expression patterns of some genes expressed in AIB interneurons

**Figure 6:**
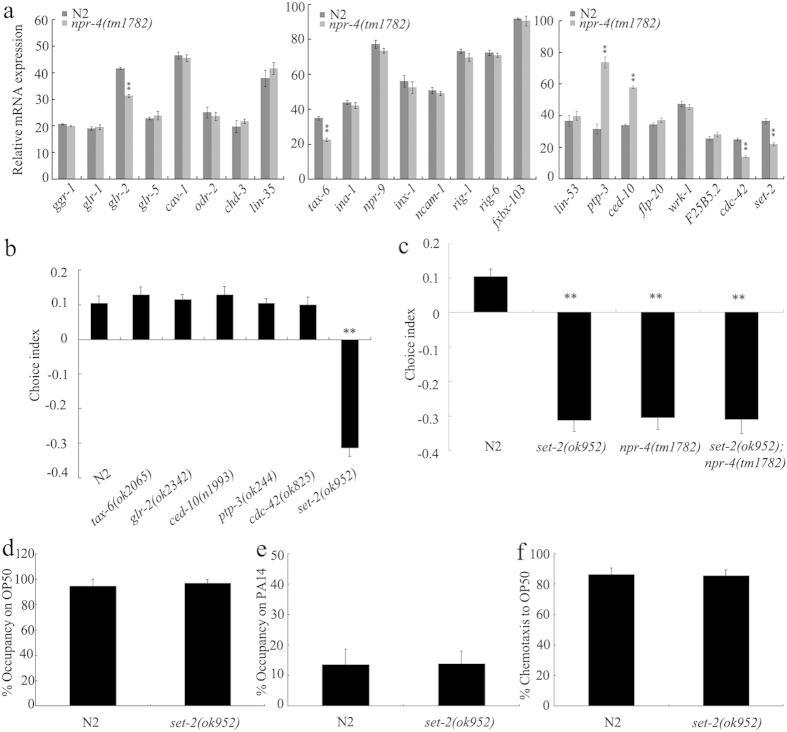
Identification of downstream target for NPR-4 in regulating preference choice in nematodes. (**a**) Mutation of *npr-4* gene altered expression patterns of some genes expressed in AIB interneurons. (**b**) Mutation of *set-2* gene induced the deficit in preference choice. (**c**) Genetic interaction of *npr-4* with *set-2* in regulating preference choice. (**d**–**f**) Leaving behavior from bacterial lawns and chemotaxis to OP50 in *set-2* mutants. Bars represent means ± S.E.M. ^**^P < 0.01 *vs* N2.

To identify candidate targeted genes for *npr-4* in regulating preference choice, we examined expression patterns of genes expressed in AIB interneurons (http://legacy.wormbase.org/db/searches/expr_search#results). Among 24 genes expressed in AIB interneurons, we found that mutation of *npr-4* gene caused the significant increase in expression levels of *ptp-3*, and *ced-10* genes, and the significant decrease in expression levels of *glr-2, tax-6, cdc-42*, and *set-2* genes (Fig. 6a). In *C. elegans, ptp-3* gene encodes a receptor-like tyrosine phosphatase, *ced-10* gene encodes a GTPase, *glr-2* gene encodes a glutamate receptor, *tax-6* gene encodes a calcineurin A, *cdc-42* gene encodes a RHO GTPase, and *set-2* gene encodes a histone H3 at lysine 4 (H3K4) methyltransferase.

### SET-2 functioned downstream of NPR-4 to regulate preference choice

We further used the available mutants for candidate targeted genes of *npr-4* to investigate their possible function in regulating preference choice in nematodes. Among 7 candidate genes examined, mutation of *set-2* gene caused the significant decrease in choice index compared with wild-type N2 (Fig. 6b). Moreover, preference choice phenotype of double mutant of *set-2(ok952);npr-4(tm1782)* was similar to that of single mutant of *set-2(ok952)* or *npr-4(tm1782)* (Fig. 6c), suggesting that SET-4 and NPR-4 may function genetically in the same pathway to regulate preference choice in nematodes. *set-2(ok952)* mutants had similar phenotypes of leaving behavior from OP50 or PA14 lawns and chemotaxis to OP50 to those in wild-type N2 (Fig. 6d–f).

### Identification of SET-2 mediated signaling pathway in regulating preference choice

**Figure 7:**
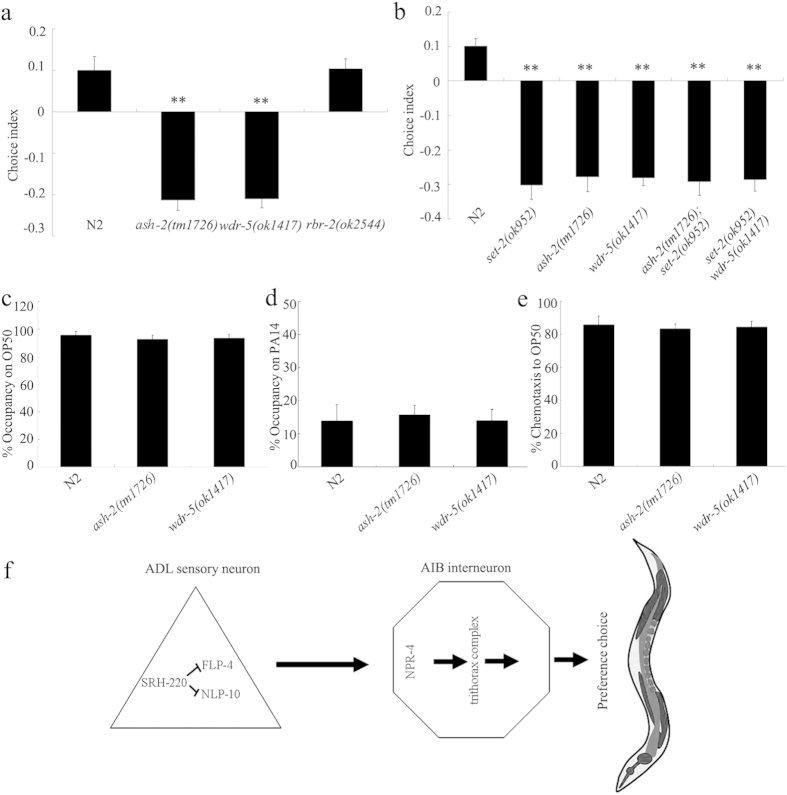
SET-2 functioned together with ASH-2 and WDR-5 to regulate preference choice in nematodes. (**a**) Preference choice phenotype in *ash-2, wdr-5*, or *rbr-2* mutants. (**b**) Genetic interaction of SET-2 with ASH-2 or WDR-5 in regulating preference choice. (**c**) Leaving behavior from bacterial lawns and chemotaxis to OP50 in *ash-2* or *wdr-5* mutants. (**d**) The molecular signals regulating preference choice in the neuronal circuit of ADL sensory neuron-AIB interneuron. Sensory neurons are shown as triangles and interneurons as hexagons. Arrows indicate chemical synapses. Bars represent means ± S.E.M. ^**^P < 0.01 *vs* N2.

In *C. elegans*, it was reported that ASH, WDR-5, and SET-2 constitute an ASH-2 trithorax complex, which trimethylates histone H3K4^32^. ASH-2 is a trithorax group protein, and WDR-5 is a WD40 repeat-containing protein. RBR-2 is a H3K4 demethylase. We found that mutation of *ash-2* or *wdr-5* gene caused the significant decrease in choice index compared with wild-type N2; however, mutation of *rbr-2* did not significantly affect preference choice behavior (Fig. 7a). These results suggest the involvement of ASH-2 trithorax complex in the control of preference choice behavior in nematodes.

In the ASH-2 trithorax complex, genetic interactions further indicate that SET-2 might function in the same pathway with ASH-2 in regulating preference choice (Fig. 7b). Similarly, SET-2 might also function in the same pathway with WDR-5 in regulating preference choice (Fig. 7b). *ash-2(tm1726)* or *wdr-5(ok1417)* mutants had similar phenotypes of leaving behavior from OP50 or PA14 lawns and chemotaxis to OP50 to those in wild-type N2 (Fig. 7d,e). Moreover, expression of *set-2, ash-2*, or *wdr-5* in AIB interneurons rescued the deficit in preference choice in corresponding mutant (Fig. S5).

## Discussion

Preference choice to food is an important behavior for animals to survival in and adapt to the environment. Preference choice on bacterial food is an important behavioral mechanism for nematodes when they are living in the environment. For the neuronal circuit of preference choice on bacterial food, it has been reported that AWB or AWC sensory neurons are involved in the control of preference choice because of their functions in affecting olfactory perception in nematodes^14^. ASH and ADL are two important sensory neurons among the amphid neurons, and have been proven to have the potentials in sensing and mediating avoidance of noxious environmental stimuli^18^,^19^,^20^. In the assayed preference choice model, two bacterial foods, OP50 and PA14, were used, and PA14 is normally considered a noxious environmental stimuli. Using strains with genetically silencing ASH or ADL sensory neurons, interestingly, we found that ASH sensory neurons were not involved in the control of preference choice on bacterial food in nematodes (Fig. 1b). In contrast, we observed that both genetically silencing and *ChR2*-mediated activation of ADL sensory neurons significantly influenced preference choice on bacterial food (Fig. 1b,c). These results suggest the crucial role of ADL sensory neurons in regulating preference choice on bacterial food in nematodes. In other words, ADL sensory neurons may regulate preference choice to bacterial food through a neuronal regulation mechanism different from AWB or AWC sensory neurons.

ARR-1, the *C. elegans* beta-arrestin ortholog, is predicted to act as an adaptor protein that potentially activates the GPCRs to regulate different biological processes such as innate immunity, and can be used as a tool to examine the possible role of GPCRs in regulating specific phenotype^23^,^33^. We further found that mutation of *arr-1* gene also noticeably affected preference choice on bacterial food (Fig. S2). In *C. elegans*, ARR-1 is expressed throughout the nervous system including the ADL sensory neurons^33^. Moreover, we observed that expression of *arr-1* in ADL sensory neurons rescued the deficits in preference choice on bacterial food in *arr-1(ok401)* mutants (Fig. S2a), implying that GPCRs in ADL sensory neurons may be involved in the control of preference choice on bacterial food in nematodes. Among the GPCRs expressed in ADL sensory neurons, we further identified the GPCR/SRH-220 as a regulator of preference choice to bacterial food. Mutation of *srh-220* led to enhanced preference choice index; however, overexpression of *srh-220* resulted in decreased preference choice index (Fig. 2).

Previous studies have suggested that the peptide signaling may play a prominent role in the control of behavioral choice, and concentrations of peptides may encode the internal states influencing behavior choice of nematodes^6^. The signaling from ADL sensory neurons may be primarily peptidergic with respect to the control of some behaviors such as modulation of ASH-mediated aversive response^27^,^28^. By analyzing the phenotype in nematodes with ADL RNAi knockdown of *unc-31, gas-1*, or *pde-4* gene, we found that ADL RNAi knockdown of *unc-31, gas-1*, or *pde-4* gene resulted in decreased preference choice index (Fig. 3), suggesting that ADL sensory neurons may also regulate preference choice behavior through the peptidergic signals in nematodes. Among the genes encoding neuropeptides expressed in ADL sensory neurons, we found that two genes, *flp-4* and *nlp-10*, were involved in the control of preference choice behavior (Fig. 4a–d), implying that the neuropeptides encoded by these genes in ADL sensory neurons may be required for the control of preference choice.

In ADL sensory neurons, we found that mutation of *flp-4* or *nlp-10* gene suppressed preference choice phenotype caused by *srh-220* gene mutation (Fig. 4e), suggesting that GPCR/SRH-220 may regulate the preference choice through influencing the function of FLP-4 or NLP-10 in nematodes. More importantly, we found that activation of ADL sensory neurons suppressed the preference choice behavior in nematodes overexpressing *srh-220* gene (Fig. 2f), suggesting that ADL activity may regulate preference choice to bacterial food by inhibiting function of GPCR/SRH-220. Therefore, our results raise a signal cascade of GPCR/SRH-220-FLP-4/NLP-10 in ADL sensory neurons involved in the control of preference choice behavior. That is, activation of ADL sensory may regulate preference choice behavior by increasing function of FLP-4 or NLP-10 through the inhibition of GPCR/SRH-220 in nematodes.

Previous studies have implied three main neuronal circuit motifs for the control of behavioral choice in nematodes^6^. These motifs involve the changes in the strength of synaptic connections or changes in the basal activity of interneutons or sensory neurons to which interneurons are electrically coupled^6^. In *C. elegans*, synaptic connections can be established between ADL sensory neurons and AIA, AIB, AVA, AVB, or AVD interneurons^3^ (Fig. 5e). Considering that mutation of *npr-4* gene encoding candidate receptor for *flp-4* resulted in the deficit in preference choice behavior (Fig. 5a–d), we investigated the neuron-specific activity of NPR-4 in regulating preference choice behavior. Interestingly, we found that expression of *npr-4* gene in AIB interneurons rescued the deficit in preference choice in *npr-4* mutants (Fig. 5f), suggesting that the released FLP-4 from ADL sensory neurons may regulate preference choice behavior through the functions of its receptor of NPR-4 in AIB interneurons.

For the underlying molecular mechanism of NPR-4 in regulating preference choice in AIB interneurons, we found that NPR-4 in AIB interneurons may regulate preference choice through the function of SET-2, a histone H3K4 methyltransferase. Several lines of evidence were raised to support this notion. Firstly, expression of *set-2* gene was altered by *npr-4* mutation (Fig. 6a). Secondly, mutation of *set-2* gene led to the decrease in choice index (Fig. 6b). Thirdly, genetic assay suggests that SET-4 and NPR-4 may function in the same pathway to regulate preference choice (Fig. 6c).

For the SET-2 mediated signaling pathway in regulating preference choice, we found that SET-2 acted in the same pathway with the other two members in the ASH-2 trithorax complex, ASH-2 and WDR-5, to regulate preference choice behavior (Fig. 7a,b). These results imply the possible pivotal role of trimethylation of histone H3K4 in the control of preference choice in nematodes. Interestingly, we did not detect the obvious involvement of RBR-2, a H3K4 demethylase, in the control of preference choice behavior (Fig. 7a). Therefore, in the neuronal circuit of “ADL sensory neuron-AIB interneuron”, the released neuropeptide of FLP-4 may regulate preference choice by activating its receptor of NPR-4 in AIB interneurons (Fig. 7f). NPR-4 may further regulate preference choice through the functions of an ASH-2 trithorax complex.

In summary, our data show that ADL sensory neurons were involved in the control of preference choice behavior. Function of ARR-1 in ADL sensory neurons suggests the potential function of GPCRs in ADL sensory neurons in the control of preference choice. In ADL sensory neurons, the GPCR/SRH-220 was identified to regulate preference choice by affecting the function of neuropeptide of FLP-4 or NLP-10. Based on the functional analysis of FLP-4 and its receptor of NPR-4, we raised a neuronal circuit of “ADL sensory neuron-AIB interneuron” involved in the control of preference choice. NPR-4 further regulated preference choice through the functions of an ASH-2 trithorax complex consisting of SET-2, ASH-2, and WDR-5, implying that NPR-4 may regulate preference choice behavior by mediating a molecular machinery for the trimethylation of histone H3K4 in nematodes.

## Methods

### *C. elegans* and bacterial strains and genetics

Nematodes were grown on nematode growth medium (NGM) plates seeded with *Escherichia coli* OP50 at 20 °C as described^34^. The following strains were used in the current study: wild-type Bristol N2, mutants of *lite-1(ce314)* X, *arr-1(ok401)* X, *srh-220(tm3939)* IV, *flp-21(ok889)* V, *nlp-7(tm1970)* X, *nlp-8(ok1799)* I, *nlp-10(tm6232)* III, *npr-4(tm1782)* X, *tax-6(ok2065)* IV, *glr-2(ok2342)* III, *ced-10(n1993)* IV, *ptp-3(ok244)* II, *cdc-42(ok825)* II, *gsa-1(ce94)* I, *pde-4(ok1290)* II, *set-2(ok952)* III, *ash-2(tm1726)* II, *wdr-5.1(ok1417)* III, and *rbr-2(ok2544)* IV, and transgenic strains of *quEx128*[*npr-9::GFP* + pRF4 *rol-6(su1006)*] expressing *npr-9* in AIB interneurons and ASH::TeTx^20^. All the used mutants were backcrossed to N2 for at least five times. Double mutant strains without additional marker mutations were constructed using standard genetic methods and verified by complementation testing. At least five independent lines were examined for each rescue experiment.

Bacterial strains used were *E. coli* OP50 and *P. aeruginosa* PA14. *E. coli* and *P. aeruginosa* cultures were grown in Luria-Bertani (LB) broth overnight at 37 °C.

### Behavioral assay

Behavioral choice was assayed as described previously^12^,^14^. Embryos collected by bleaching were grown on a standard NGM plate fed with *E. coli* OP50. All the behavioral experiments were performed with young adults maintained at 20 °C. Behavioral choice assay was performed on 9-cm NGM plates. Bacterial food with a diameter of 0.5-cm was seeded at the distance of 1.5 cm from periphery (Fig. 1a). Animals were then put on the center of the assay plates and allowed to migrate. Number of nematodes on each bacterial lawn was counted after treatment for 2 h at 20 °C. Behavioral choice index was calculated as (PA14 number – OP50 number)/total number. In this behavioral choice assay system, choice index of −1.0 represents the complete preference for OP50, choice index of 1.0 represents the complete preference for PA14, and choice index of 0 represents an equal distribution. Ten replicates were performed.

The method for assay of leaving behavior from OP50 or PA14 lawn was performed as described^35^. Small lawns of PA14 or OP50 were cultured on 6-cm NGM plates overnight at 25 °C, and 20 young adults were put in the center of each bacteria lawn. The number of animals on each lawn was counted after 16 h. Ten replicates were performed.

To analyze the chemotaxis perception of nematodes to OP50, OP50 with a diameter of 0.5-cm was seeded at one side with the distance of 1.5 cm from periphery on 9-cm NGM plates. M9 buffer was added to the other side with the distance of 1.5 cm from periphery on 9-cm NGM plates. Nematodes were put on the center of the assay plates and allowed to migrate for 2 h. The chemotaxis index was calculated as (OP50 number – M9 buffer number)/total number. Ten replicates were performed.

### DNA constructs and germline transformation

To generate entry vectors carrying promoter sequences, promoter regions were amplified by polymerase chain reaction (PCR) from wild-type *C. elegans* genomic DNA (1.6 kb for *srh-220* promoter used for ADL-specific expression^26^, 2.8 kb for *gcy-28.d* promoter used for AIA-specific expression, 1.9 kb for *npr-9* promoter used for AIB-specific expression, and 1.8 kb for *pept-3* promoter used for AVA-specific expression, and then inserted into the pPD95_77 vector in the sense orientation. *egl-1, arr-1, srh-220, npr-4, set-2, ash-2*, or *wdr-5* cDNA was amplified by PCR. The sequences of the amplified cDNA were verified by sequencing, and then cDNA was inserted into corresponding entry vectors carrying promoter sequence. To construct P*srh-220-ChR2* and P*npr-9-ChR2* for optical activation of ADL sensory neurons or AIB interneurons, the *srh-220* or *npr-9* promoter fragment was inserted into the 95_75-*ChR2* vector. To determine whether *npr-4* is expressed in AIB interneurons, the *npr-4* promoter (2.7 kb) was inserted into 95_77-mcherry vector and then transformed into the strain of *quEx128*. Germline transformation was performed as described^36^ by coinjecting the testing DNA at a concentration of 10–40 μg/mL and the marker DNA of P*dop-1::rfp* or P*lin-44::gfp* at a concentration of 60 μg/mL into the gonad of nematodes.

### Optical genetic assay

Light-activated ChR2, a directly light-gated cation channel from the green alga *Chlamydomonas reinhardtii*, can utilize chromophore ATR to enable the activity of specific neurons in *C. elegans*^37^. The *srh-220* promoter was used for the ADL-specific expression, and the *npr-9* promoter was used for the AIB-specific expression. Nematodes expressing *ChR2* in ADL sensory neurons or AIB interneurons were grown on OP50-seeded NGM agar plates containing 50 μM of ATR. Plates were seeded on day 0. Nematodes were transferred to OP50-seeded plates containing 50 μM of ATR on day 1 in the dark. After starvation for 5 h, nematodes were transferred to assay plates containing 50 μM of ATR for behavioral analysis on day 2. During the assay, whole field illumination was performed, and *ChR2* was excited by a round blue light in diameter of 9.5 cm sourced from LED array (460–470 nm, ~0.5 mW/mm^2^) constructed in a LED light source (Chenyufanli Trading Co. LTD., Nanjing, China). Light intensity measured at the sample was 5 mW/mm^2^ of 465 nm light. For optogenetic experiments, light intensity was monitored using an optical power meter (PM100, Thorlabs). All work was carried out under low-illumination conditions to prevent the preactivation of *ChR2*-expressing neurons. Nematodes without ATR treatment were used as the control. Optogenetical activation was performed under the *lite-1* mutation background. Ten replicates were performed.

### RNAi

RNAi was performed by feeding nematodes with *E. coli* strain HT115 (DE3) expressing double-stranded RNA that is homologous to a target gene as described^38^. *E. coli* HT115 (DE3) grown in LB broth containing ampicillin (100 μg/ml) at 37 °C overnight was plated onto NGM containing 100 μg/mL ampicillin and 5 mM isopropyl 1-thio-β-D-galactopyranoside (IPTG). L2 larvae were placed on RNAi or vector control plates for 2 days at 20 °C until nematodes became gravid. Gravid adults were transferred to fresh RNAi-expressing bacterial lawns and allowed to lay eggs for 2 h to obtain the second generation of RNAi population. Eggs were allowed to develop at 20 °C to young adults for the subsequent assays. *unc-22* RNAi was included as a positive control.

### ADL neuron-specific RNAi

The method was performed basically as described^39^. The exon rich fragment of genomic sequence of *flp-4, pde-4, unc-31*, or *gsa-1* gene was amplified by PCR to yield product A. The *srh-220* promoter was used for ADL-specific expression, and amplified in two different reactions with promoter reverse sense primer or promoter reverse antisense primer together with promoter forward primer to yield product B and C. Product A was further fused by amplification to B or C using the nested primers to yield fragments D and E. In fragment D, the *flp-4, pde-4, unc-31*, or *gsa-1* gene was transcribed by ADL-specific promoter in the sense orientation. In fragment E, the *flp-4, pde-4, unc-31*, or *gsa-1* gene was transcribed by the ADL-specific promoter in the antisense orientation. The fragments D and E were mixed in equimolar amounts and injected at 25–100 ng/μL into the wild-type nematodes. The P*lin-44::gfp* was used as a transgenic marker.

### Reverse transcription and quantitative real-time PCR (qRT-PCR)

Total RNA of nematodes was extracted using RNeasy Mini Kit (Qiagen, Valencia, Ca, USA). Purity and concentration of RNA were evaluated by OD260/280 in a spectrophotometer. Total RNA was reverse-transcribed using cDNA synthesis kit (Bio-Rad Laboratories, Hercules, CA, USA). After cDNA synthesis, real-time PCR was performed using SYBR Premix Ex Taq™ (Takara) for amplification of the PCR products. The *act-1* gene was chosen as a reference gene. Relative quantification of targeted genes in comparison to reference *act-1* gene was determined, and the final results were expressed as relative expression ratio between targeted gene and reference gene. The primer information was shown in Table S1. Three replicates were performed.

### Statistical analysis

All data in this article were expressed as means ± standard error of the mean (S.E.M.). Graphs were generated using Microsoft Excel (Microsoft Corp., Redmond, WA). Statistical analysis was performed using SPSS 12.0 (SPSS Inc., Chicago, USA). Differences between groups were determined using analysis of variance (ANOVA). The probability levels of 0.05 and 0.01 were considered to be statistically significant.

## Additional Information

**How to cite this article**: Yu, Y. *et al*. FLP-4 neuropeptide and its receptor in a neuronal circuit regulate preference choice through functions of ASH-2 trithorax complex in *Caenorhabditis elegans. Sci. Rep.* **6**, 21485; doi: 10.1038/srep21485 (2016).

## Supplementary Material

Supplementary Information

## References

[b1] Rangel A., Camerer C. & Montague P. R. A framework for studying the neurobiology of value-based decision making. Nat. Rev. Neurosci. 9, 545–556 (2008). 18545266 10.1038/nrn2357 PMC4332708

[b2] Palmer C. R. & Kristan W. B. Jr. Contextual modulation of behavioral choice. Curr. Opin. Neurobiol. 21, 520–526 (2011). 21624826 10.1016/j.conb.2011.05.003 PMC3478953

[b3] White J. G., Southgate E., Thomson J. N. & Brenner S. The structure of the nervous system of *Caenorhabditis elegans*. Philos. Trans. R. Soc. Lond. B Biol. Sci. 314, 1–340 (1986). 22462104 10.1098/rstb.1986.0056

[b4] Bargmann C. I. Chemosensation In C. elegans. Wormbook. 1–29 (2006). 18050433 10.1895/wormbook.1.123.1 PMC4781564

[b5] Ye H.–Y., Ye B.–P. & Wang D.-Y. Learning and learning choice in the nematode *Caenorhabditis elegans*. Neurosci. Bull. 22, 355–360 (2006). 17690721

[b6] Faumout S., Lindsay T. H. & Lockery S. R. Neuronal microcircuits for decision making In C. elegans. Curr. Opin. Neurobiol. 22, 580–591 (2012). 22699037 10.1016/j.conb.2012.05.005 PMC3593597

[b7] Li Y.–X., Wang Y., Hu Y.–O., Zhong J.–X. & Wang D.-Y. Modulation of the assay system for the sensory integration of 2 sensory stimuli that inhibit each other in nematode *Caenorhabditis elegans*. Neurosci. Bull. 27, 69–82 (2011). 21441968 10.1007/s12264-011-1152-z PMC5560346

[b8] Wang D.–Y., Yu Y.–L., Li Y.–X., Wang Y. & Wang D.-Y. Dopamine receptors antagonistically regulate behavioral choice between conflicting alternatives In C. elegans. PLoS ONE 9, e115985 (2014). 25536037 10.1371/journal.pone.0115985 PMC4275273

[b9] Ishihara T. . HEN-1, a secretary protein with an LDL receptor motif, regulates sensory integration and learning in *Caenorhabditis elegans*. Cell 109, 639–649 (2002). 12062106 10.1016/s0092-8674(02)00748-1

[b10] Kawano T. . An imbalancing act: gap junctions reduce the backward motor circuit activity to bias *C. elegans* for forward locomotion. Neuron 72, 572–586 (2011). 22099460 10.1016/j.neuron.2011.09.005

[b11] Matsuura T., Oikawa T., Wakabayashi T. & Shingai R. Effect of simultaneous presentation of multiple attractants on chemotactic responses of the nematode *Caenorhabditis elegans*. Neurosci. Res. 48, 419–429 (2004). 15041195 10.1016/j.neures.2003.12.008

[b12] Zhang Y., Lu H. & Bargmann C. I. Pathogenic bacteria induce aversive olfactory learning in Caenorhabditis elegans. Nature 438, 179–184 (2005). 10.1038/nature04216 16281027

[b13] Bargmann C. I., Hartwieg E. & Horvitz H. R. Odorant-selective genes and neurons mediate olfaction In C. elegans. Cell 74, 515–527 (1993). 8348618 10.1016/0092-8674(93)80053-h

[b14] Abada E. A. . *C. elegans* behavior of preference choice on bacterial food. Mol. Cells 28, 209–213 (2009). 19756391 10.1007/s10059-009-0124-x

[b15] Meisel J. & Kim D. H. Behavioral avoidance of pathogenic bacteria by *Caenorhabditis elegans*. Trends Immunol. 35, 465–470 (2014). 25240986 10.1016/j.it.2014.08.008

[b16] Ha H. I. . Functional organization of a neural network for aversive olfactory learning in *Caenorhabditis elegans*. Neuron 68, 1173–1186 (2010). 21172617 10.1016/j.neuron.2010.11.025 PMC3038580

[b17] Harris G. . Dissecting the signaling mechanism underlying recognition and preference of food odors. J. Neurosci. 34, 9389–9403 (2014). 25009271 10.1523/JNEUROSCI.0012-14.2014 PMC4087214

[b18] Bargmann C. I., Thomas J. H. & Horvitz H. R. Chemosensory cell function in the behavior and development of *Caenorhabditis elegans*. Cold Spring Harb. Symp. Quant. Biol. 55, 529–538 (1990). 2132836 10.1101/sqb.1990.055.01.051

[b19] Sambongi Y. . *Caenorhabditis elegans* senses protons through amphid chemosensory neurons: proton signals elicit avoidance behavior. Neuroreport 11, 2229–2232 (2000). 10923676 10.1097/00001756-200007140-00033

[b20] Guo M. . Reciprocal inhibition between sensory ASH and ASI neurons modulates nociception and avoidance in *Caenorhabditis elegans*. Nat. Commun. 6, 5655 (2015). 25585042 10.1038/ncomms6655

[b21] Styer K. L. . Innate immunityy in *Caenorhabditis elegans* is regulated by neurons expressing NPR-1/GPCR. Science 322, 460–464 (2008). 18801967 10.1126/science.1163673 PMC2831475

[b22] Schmitt C., Schultheis C., Husson S. J., Liewald J. F. & Gottschalk A. Specific expression of channelrhodopsin-2 in single neurons of *Caenorhabditis elegans*. PLoS ONE 7, e43164 (2012). 22952643 10.1371/journal.pone.0043164 PMC3431400

[b23] Singh V. & Aballay A. Endoplasmic reticulum stress pathway required for immune homeostasis is neurally controlled by Arrestin-1. J. Biol. Chem. 287, 33191–33197 (2012). 22875856 10.1074/jbc.M112.398362 PMC3460425

[b24] Troemel E. R., Chou J. H., Dwyer N. D., Colbert H. A. & Bargmann C. I. Divergent seven transmembrane receptors are candidate chemosensory receptors in *C. elegans*. Cell 83, 207–218 (1995). 7585938 10.1016/0092-8674(95)90162-0

[b25] McCarroll S. A., Li H. & Bargmann C. I. Identification of transcriptional regulatory elements in chemosensory receptor genes by probabilistic segmentation. Curr. Biol. 15, 347–352 (2005). 15723796 10.1016/j.cub.2005.02.023

[b26] Mohan S., Timbers T. A., Kennedy J., Blacque O. E. & Leroux M. R. Striated rootlet and nonfilamentous forms of rootletin maintain ciliary function. Curr. Biol. 23, 2016–2022 (2013). 24094853 10.1016/j.cub.2013.08.033

[b27] Harris G. . The monoaminergic modulation of sensory-mediated aversive responses in *Caenorhabditis elegans* requires glutamatergic/peptidergic cotransmission. J. Neurosci. 30, 7889–7899 (2010). 20534837 10.1523/JNEUROSCI.0497-10.2010 PMC3005568

[b28] Mills H. . Monoamines and neuropeptides interact to inhibit aversive behaviour in *Caenorhabditis elegans*. EMBO J. 31, 667–678 (2012). 22124329 10.1038/emboj.2011.422 PMC3273394

[b29] Zhou K. M. . PKA activation bypasses the requirement for UNC-31 in the docking of dense core vesicles from *C. elegans* neurons. Neuron 56, 657–669 (2007). 18031683 10.1016/j.neuron.2007.09.015

[b30] Li C. & Kim K. Neuropeptides, *WormBook*, ed. The *C. elegans* Research Community, WormBook, doi: 10.1895/wormbook.1.142.1 (2008).

[b31] Cohen M. . Coordinated regulation of foraging and metabolism in *C. elegans* by RFamide neuropeptide signaling. Cell Metab. 9, 375–385 (2009). 19356718 10.1016/j.cmet.2009.02.003

[b32] Greer E. L. . Members of the H3K4 trimethylation complex regulate lifespan in a germline-dependent manner in *C. elegans*. Nature 466, 383–387 (2010). 20555324 10.1038/nature09195 PMC3075006

[b33] Palmitessa A. . *Caenorhabditis elegans* arrestin regulate neural G protein signaling and olfactory adaptation and recovery. J. Biol. Chem. 280, 24649–24662 (2005). 15878875 10.1074/jbc.M502637200

[b34] Brenner S. The genetics of *Caenorhabditis elegans*. Genetics 77, 71–94 (1974). 4366476 10.1093/genetics/77.1.71 PMC1213120

[b35] Pradel E. . Detection and avoidance of a natural product from the pathogenic bacterium *Serratia marcescens* by *Caenorhabditis elegans*. Proc. Natl. Acad. Sci. USA 104, 2295–2300 (2007). 17267603 10.1073/pnas.0610281104 PMC1892944

[b36] Mello C. & Fire A. DNA transformation. Methods Cell. Biol. 48, 451–482 (1995). 8531738

[b37] Nagel G. . Light activation of channelrhodopsin-2 in excitable cells of *Caenorhabditis elegans* triggers rapid behavioral responses. Curr. Biol. 15, 2279–2284 (2005). 16360690 10.1016/j.cub.2005.11.032

[b38] Kamath R. K., Martinez-Campos M., Zipperlen P., Fraser A. G. & Ahringer J. Effectiveness of specific RNA-mediated interference through ingested double stranded RNA in *C. elegans*. Genome Biol. 2, 1–10 (2001). 10.1186/gb-2000-2-1-research0002 PMC17598 11178279

[b39] Esposito G., Di Schiavi E., Bergamasco C. & Bazzicalupo P. Efficient and cell specific knock-down of gene function in targeted *C. elegans* neurons. Gene 395, 170–176 (2007). 17459615 10.1016/j.gene.2007.03.002

