# Supplementary material for: FLP-4 neuropeptide and its receptor in a neuronal circuit regulate preference choice through functions of ASH-2 trithorax complex in Caenorhabditis elegans
Source: Sci Rep. 2016 Feb 18;6:21485. doi: 10.1038/srep21485 (PMC4757837; doi:10.1038/srep21485)
Supplement: Supplementary Information [file srep21485-s1.doc]

**FLP-4 neuropeptide and its receptor in a neuronal circuit regulate preference choice through functions of ASH-2 trithorax complex in *Caenorhabditis elegans***

Yonglin Yu, Lingtong Zhi, Xiangmin Guan, Daoyong Wang, Dayong Wang^*^

Key Laboratory of Developmental Genes and Human Disease in Ministry of Education, Medical School, Southeast University, Nanjing 210009, China

**Supporting Information:**

**
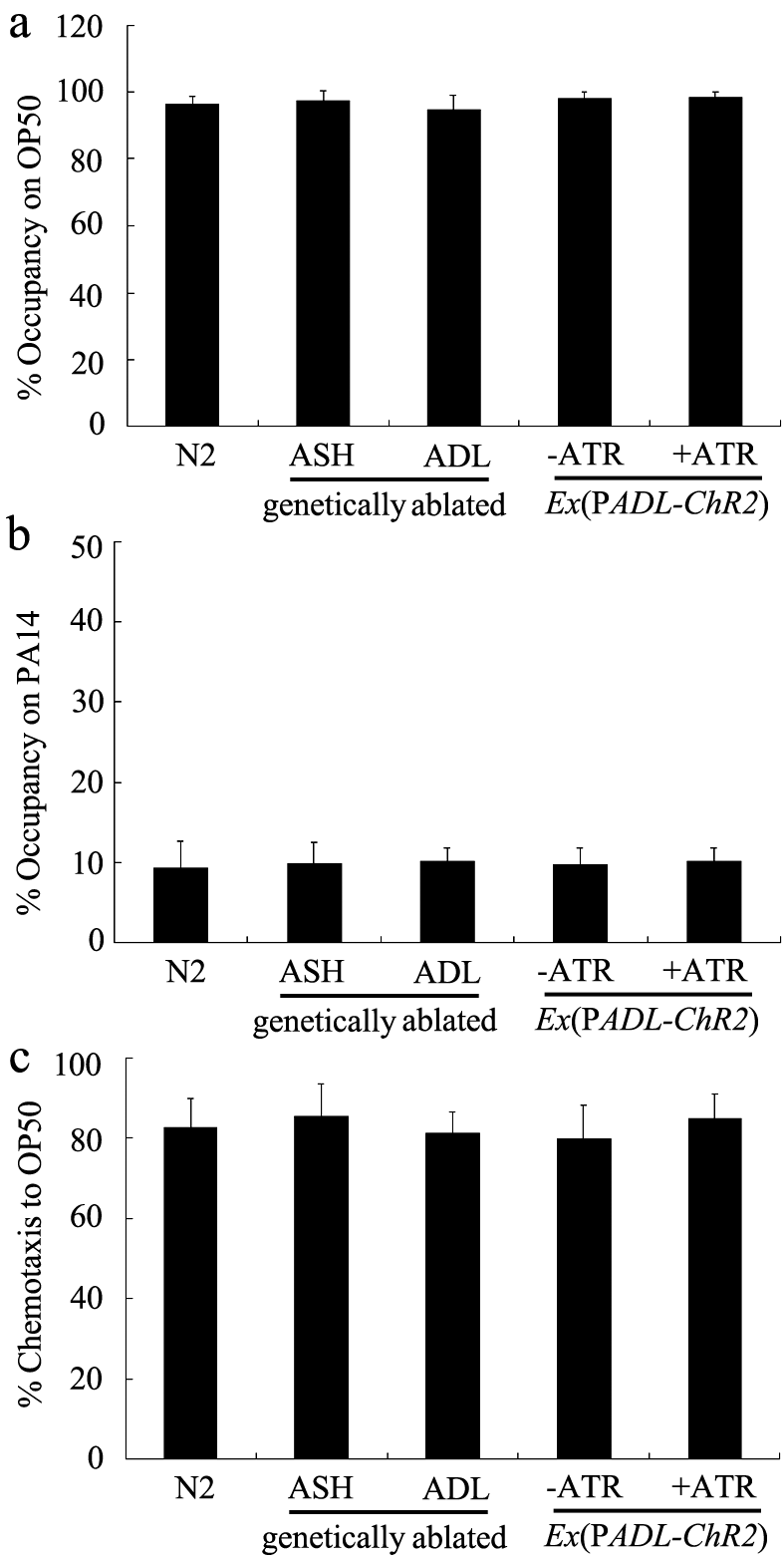
**

**Figure S1 | Leaving behavior from bacterial lawns (a-b) and chemotaxis to OP50 (c) for nematodes with genetically ablated or optogenetically activating ADL sensory neurons.** Bars represent means ± S.E.M.

**
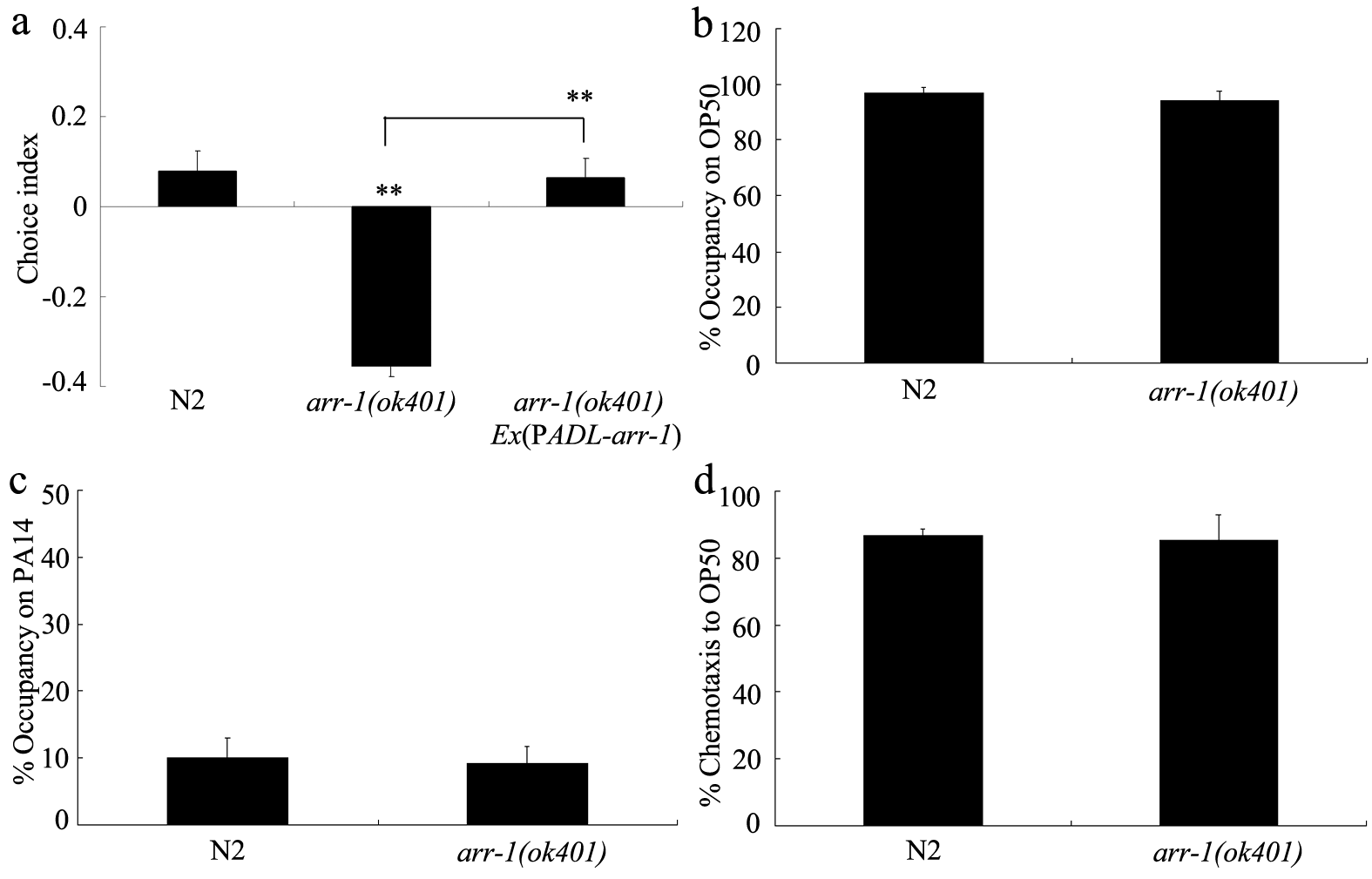
**

**Figure S2 | ARR-1 expressing in ADL sensory neurons was required for the control of preference choice in nematodes.** (a) Expression of *arr-1* gene in ADL sensory neurons rescued the deficit in preference choice in *arr-1* mutants. (b-d) Leaving behavior from bacterial lawns and chemotaxis to OP50 of *arr-1* mutants. Bars represent means ± S.E.M. ^**^*P* < 0.01 *vs* N2 (if not specifically indicated).


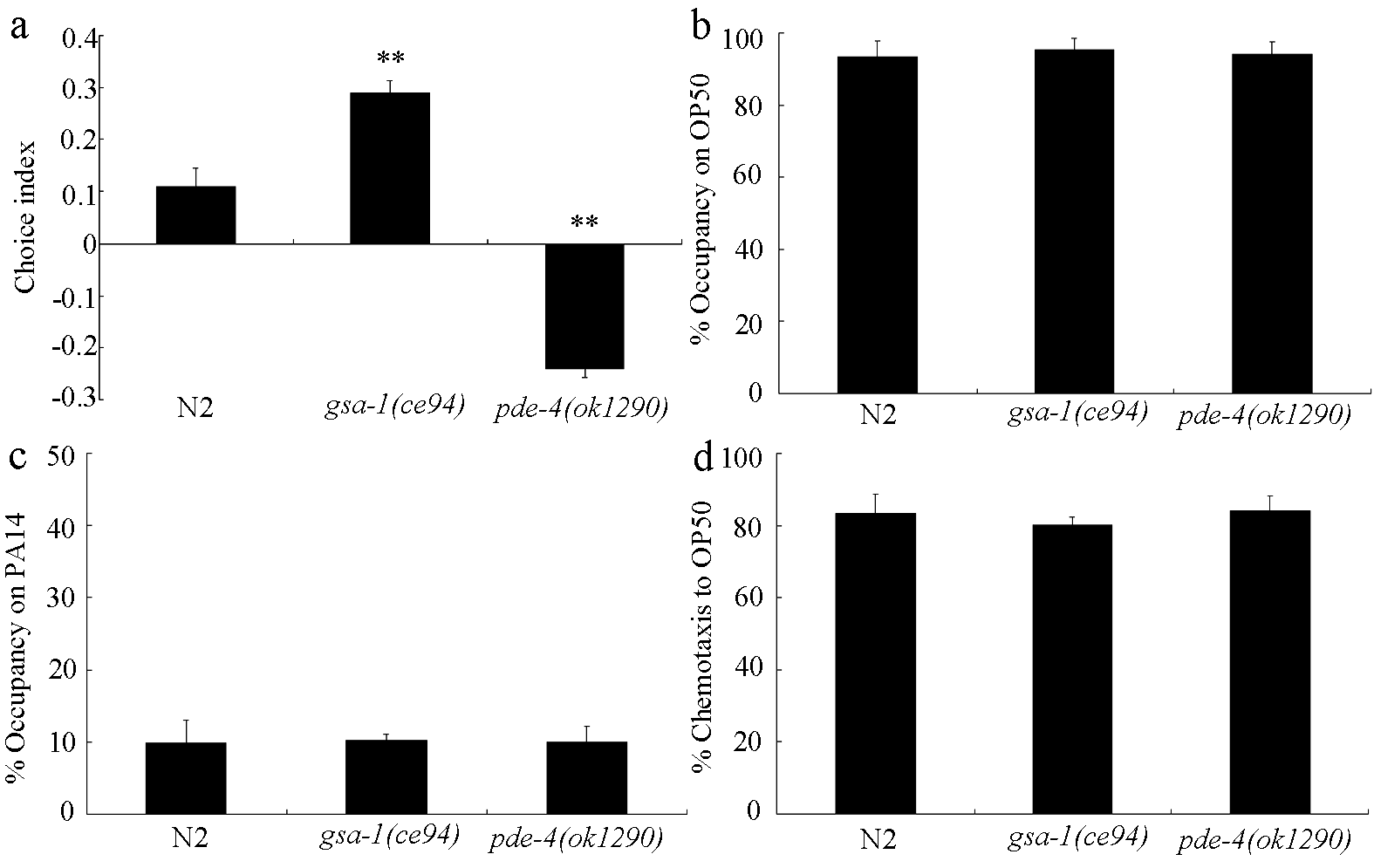


**Figure S3 | Effect of *gsa-1* or *pde-4* mutation on preference choice.**  (a) Preference choice of nematodes with mutation of *gsa-1* or *pde-4* gene. (b-d) Leaving behavior from bacterial lawns and chemotaxis to OP50 of nematodes with mutation of *gsa-1* or *pde-4* gene. Bars represent means ± S.E.M. ^**^*P* < 0.01 *vs* N2.


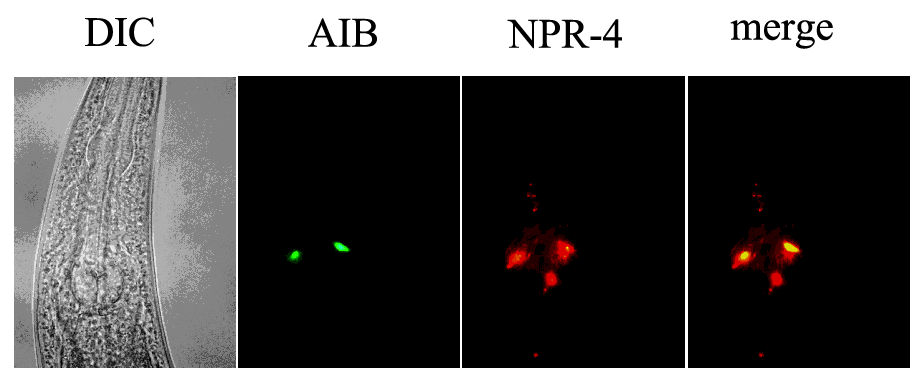


**Figure S4 | NPR-4 was expressed in AIB interneurons.** To determine whether *npr-4* is expressed in AIB interneurons, the *npr-4* promoter was inserted into 95_77-mcherry vector and then transformed into the strain of *quEx128*[*npr-9::GFP* + pRF4 *rol-6(su1006)*] expressing *npr-9* in AIB interneurons.


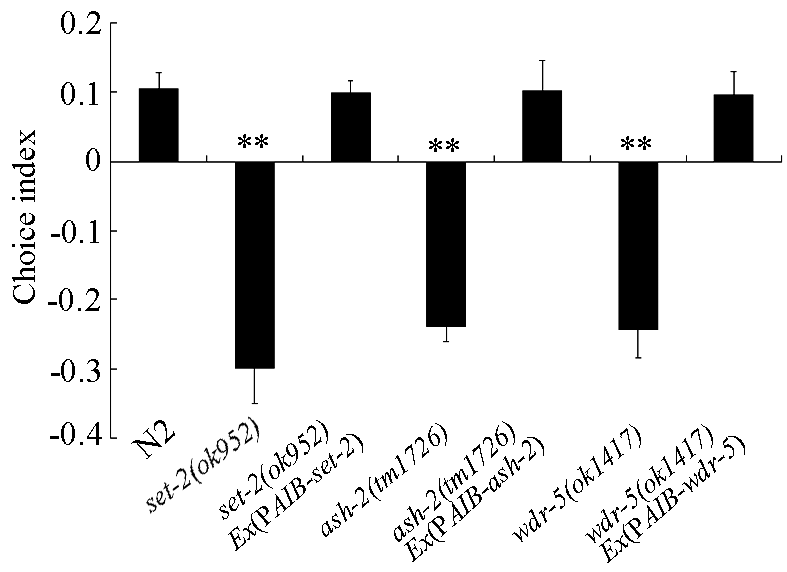


**Figure S5 | Expression of *set-2, ash-2*, or *wdr-5* gene in AIB interneurons rescued the deficit in preference choice in corresponding mutant.**  Bars represent means ± S.E.M. ^**^*P* < 0.01 *vs* N2.

**Table S1 | Primers used for quantitative real-time polymerase chain reaction (PCR)**

| Gene | Forward primer | Reverse primer |
| --- | --- | --- |
| *act-1* | CTGCAGATGTGTGACGACGAGGTT | CTGCAGGAAGCACTTGCGGTGAAC |
| *ggr-1* | TCCGGCGATCACTACTTC | CTCCTCGACTCGGTTTATTC |
| *glr-1* | CAACCAGGATCTCAATCTCA | TGCAGCTACCTGTTGTCT |
| *glr-2* | ATAAATATGGGTCAAGACAAGC | AACCAGGACACGAGGAAA |
| *glr-5* | CGCGTCTTCTTGGAGGTA | TCAGGACAGCAGCTAAGTTT |
| *cav-1* | ACGCCAACTCGATGAACA | ACGCAATCCCATGAGTGCT |
| *odr-2* | TTCGGCTCCTTCTCTTCTG | GTGGATGCTGATCTTTCCAATA |
| *chd-3* | CAAAGCTCCTCGTTTACCA | CGAGCCACCTTCTCTTCATT |
| *lin-35* | GCCAATTCTTTGCTGGTCTA | TTTCGGGATTCAGTTGTTCT |
| *tax-6* | TGCGATTCGGGACATCTTAT | TGTTGGTGGCATTCTTTCA |
| *ina-1* | GTGATGGACGACCTAGAGAA | TGGCAAATGACGGTGAAAC |
| *npr-9* | GCCGGGTAACTCTCAATCT | GACGAATACTTGAACCACTATCA |
| *inx-1* | AATGGGTGCCGTTTGTGTTA | TATGCACGGGATGTAGAA |
| *ncam-1* | ACAGAAGCCTCAAACGAATCAA | TGTCTCCTGCTCCTCACT |
| *rig-1* | ACTCGGGTGACTCGGATAAG | CGTCCAAGTTTCCGTACAAAT |
| *rig-6* | GAGGCATCGGTCCTATCAG | TCGGAGCTTCCAGTTGTA |
| *fbxb-103* | TCGAGTGCTCTTCCACATA | CACCAGATTCCGGGAGTTG |
| *lin-53* | AGTGTGGCAAATGGCTGATA | CGGCTGGAGTTTCTTCGT |
| *ptp-3* | TACAAACTGAAGAGCAATACATC | GTTCACCGCATCAAGGAC |
| *ced-10* | TGCAAGCGATCAAATGTGTC | TGCGTTTGTGGTGTAGGA |
| *flp-20* | TGTGGTTATTACACTTCTACTG | GCAACCCTTCCTCTTCAT |
| *wrk-1* | GCTTTGCCACCGATTAGA | GACACTGTTGCAGTCCTC |
| *F25B5.2* | TCGTCCTCCGACACTTCT | CAACAGCCACGACATCAA |
| *cdc-42* | CTGCTGGACAGGAAGATTAC | GTCGGTCTGTGGATACGAT |
| *set-2* | GCATCCGACTGCAATCTTC | CGTTGGAGCAATCTCATATCTT |
| *flp-4* | TTGCACTCACAGCAGCTCAT | CCGTTTGATGATCGTAATCCAC |
| *nlp-10* | CACAGCACAGAAAGCAGACG | ACCTGAGAACGGCATTGTTGA |
